# Downregulation of Type II Diabetes Mellitus and Maturity Onset Diabetes of Young Pathways in Human Pancreatic Islets from Hyperglycemic Donors

**DOI:** 10.1155/2014/237535

**Published:** 2014-10-14

**Authors:** Jalal Taneera, Petter Storm, Leif Groop

**Affiliations:** Department of Clinical Sciences, Diabetes & Endocrinology, Lund University Diabetes Center, Skåne University Hospital, Lund University, 20502 Malmö, Sweden

## Abstract

Although several molecular pathways have been linked to type 2 diabetes (T2D) pathogenesis, it is uncertain which pathway has the most implication on the disease. Changes in the expression of an entire pathway might be more important for disease pathogenesis than changes in the expression of individual genes. To identify the molecular alterations in T2D, DNA microarrays of human pancreatic islets from donors with hyperglycemia (*n* = 20) and normoglycemia (*n* = 58) were subjected to Gene Set Enrichment Analysis (GSEA). About 178 KEGG pathways were investigated for gene expression changes between hyperglycemic donors compared to normoglycemic. Pathway enrichment analysis showed that type II diabetes mellitus (T2DM) and maturity onset diabetes of the young (MODY) pathways are downregulated in hyperglycemic donors, while proteasome and spliceosome pathways are upregulated. The mean centroid of gene expression of T2DM and MODY pathways was shown to be associated positively with insulin secretion and negatively with HbA1c level. To conclude, downregulation of T2DM and MODY pathways is involved in islet function and might be involved in T2D. Also, the study demonstrates that gene expression profiles from pancreatic islets can reveal some of the biological processes related to regulation of glucose hemostats and diabetes pathogenesis.

## 1. Introduction

T2D is a multifactorial disease characterized by increased blood glucose level due to both a defect in insulin secretion from pancreatic beta-cells and impaired insulin action at the target cells. The disease is estimated to affect more than 350 million people in 2020 worldwide (http://www.idf.org/diabetesatlas/) and to contribute to other diseases such as atherosclerotic vascular disease, blindness, and kidney failure [1].

Several molecular pathways have been implicated in the disease process: insulin receptor signalling [2], carbohydrate metabolism [3], ER stress related pathway [4], cytokine signalling [5], exocytosis [6], and oxidative phosphorylation [7, 8]. However, it is unclear which of these or other pathways are disturbed in and might be responsible for T2D in its common form.

DNA microarrays expression analysis enables scientists to investigate the altered transcript levels in particular tissue from individuals with specific diseases. For example, mRNA expression profiles are generated from thousands of genes from samples of one of two classes such as cancer [9]. The differential expressed genes between classes can be ranked based on their differentiation. However, the remaining challenge is how to interpret a given list of genes into biological mechanism.

Mootha et al. have developed a statistical methodology called Gene Set Enrichment Analysis (GSEA) to define whether a given gene set is significantly enriched in a list of genes ranked by their correlation with a phenotype of interest [8, 10]. GSEA has been shown to have an increased capacity to detect modest but coordinated changes in prespecified set of related genes. GSEA has been successfully used to uncover altered metabolic pathways in several applications such as human diabetic muscle [8], comparing mouse models of cancer with human tumors using gene-expression profiling [11], lung cancer [10], characterization of acute megakaryoblastic leukemia [12], and interaction between mRNA and miRNA in HIV-mediated neurodegeneration [13] and comparing whole blood gene expression profiling from lean and obese individuals [14].

Here, we employed GSEA to determine whether the 178 selected KEGG pathways are altered between islet gene expression from donors with normoglycemia and hyperglycemia. Pathway enrichment analysis showed that MODY and T2DM pathways are downregulated in hyperglycemic islets. The mean centroid of gene expression of T2DM and MODY pathways was shown to be significantly associated with insulin secretion and HbA1c level, which highlight that these pathways are involved in islet function.

## 2. Materials and Methods

### 2.1. Human Pancreatic Islets

Islets from cadaver donors (78 donors) were provided by the Nordic Islet Transplantation Program (www.nordicislets.org), Uppsala University. All procedures were approved by the ethics committees at Uppsala and Lund Universities. Islets were obtained from 68 nondiabetic donors (30 females, 37 males, age 59 ± 10, BMI 25.9 ± 3.5, HbA1c 5.5 ± 1.1, and days of culture 3.5 ± 1.9) and 10 T2D donors (4 females, 6 males, age 60.7 ± 12, BMI 28.1 ± 4.5, HbA1c 7.1 ± 1.2, and days of culture 2 ± 0.9). Purity of the islet preparations was assessed by dithizone staining, insulin content, and contribution of exocrine and endocrine tissue as previously described [15]. The islets were cultured in CMRL 1066 (ICN Biomedicals, Costa Mesa, CA, USA) supplemented with 10 mM/L HEPES, 2 mM/L l-glutamine, 50 *μ*g/mL gentamicin, 0.25 *μ*g/mL Fungizone (GIBCO, BRL, Gaithersburg, MD, USA), 20 *μ*g/mL ciprofloxacin (Bayer Healthcare, Leverkusen, Germany), and 10 mM/L nicotinamide at 37°C (5% CO_2_) prior to RNA preparation.

### 2.2. Microarray Gene Expression in Human Pancreatic Islets

RNA was isolated with the AllPrep DNA/RNA Mini Kit (Qiagen, Hilden, Germany). RNA quality and concentration were measured using an Agilent 2100 bioanalyzer (Bio-Rad, Hercules, CA, USA) and Nanodrop ND-1000 equipment (NanoDrop Technologies, Wilmington, DE, USA). The microarrays (GeneChip Human Gene 1.0 ST) were performed using the Affymetrix standard protocol as previously described [15]. The array data were summarized and normalized with robust multiarray analysis (RMA) method using the oligo package from BioConductor. Also, batch correction was done with COMBACT function from SVA package from BioConductor. All data are MIAME compliant, and the raw data have been deposited in a MIAME database (GEO, accession number: GSE 50398 and GSE 50397).

### 2.3. Glucose-Stimulated Insulin Secretion

Islets were hand-picked under a stereomicroscope and preincubated for 30 min at 37°C in Krebs Ringer bicarbonate (KRB) buffer (pH 7.4) containing (in mM) 120 NaCl, 25 NaHCO_3_, 4.7 KCl, 1.2 MgSO_4_, 2.5 CaCl_2_, 1.2 KH_2_PO, 10 HEPES supplemented with 0.1% bovine serum albumin, N-2 hydroxyethylpiperazine-N′-2-ethanesulfonic acid (10 mmol/L), and 1 mmol/L glucose. Each incubation vial contained 12 islets in 1.0 mL KRB buffer solution and was treated with 95% O_2_-5% CO_2_ to obtain constant pH and oxygenation. After preincubation, the buffer was changed to a KRB buffer containing either 1 mM (basal secretion) or 16.7 mM glucose (stimulated secretion). The islets were then incubated for 1 h at 37°C in a metabolic shaker (30 cycles per min). Immediately after incubation, an aliquot of the medium was removed for analysis of insulin using a radioimmunoassay kit (Euro-Diagnostica, Malmö, Sweden). Insulin content in homogenized human islets was assessed by ELISA (Mercodia, Uppsala, Sweden) and values were normalized to the total DNA in each sample as determined by a fluorometric assay (Quant-iT PicoGreen, Invitrogen Molecular Probes, Stockholm, Sweden).

### 2.4. Gene Set Enrichment Analysis (GSEA)

The GSEA software tool (version 2.0.13, www.broadinstitute.org/gsea/) was used to identify KEGG pathways (MSigDB, version 4.0) that show an overrepresentation of up- or downregulated genes between donors with hyperglycemia (HbA1c > 6%, *N* = 20) and normoglycemia (HbA1c < 6%, *N* = 30). Briefly, an enrichment score was calculated for each gene set (i.e., KEGG pathway) by ranking each gene by their expression difference using Kolmogorov-Smirnov statistic, computing a cumulative sum of each ranked in each gene set, and recording the maximum deviation from zero as the enrichment score.

### 2.5. Statistical Analysis

Data are presented as means ± S.D. Differences in expression levels were analyzed by Student's *t*-test or nonparametric Mann-Whitney tests. Correlation tests were analyzed using nonparametric Spearman's tests. The mean centroid represents the normalized gene expression levels of all genes from all individuals in the analysis with a mean of 0 and a variance of 1. All statistical tests were performed using the Statistical Package for the Social Sciences (SPSS) version 19.0 software (SPSS, Chicago, IL, USA).

## 3. Results

In this study, we used human islet microarray expression data obtained from 78 donors. The donors were subdivided into 58 normoglycemic donors with HbA1c level <6% and 20 hyperglycemic donors with HbA1c level >6% (Table 1). Normalized expression microarray data were subjected to pathway analysis of GSEA algorithm using 178 Kyoto Encyclopedia for Genes and Genomes (KEGG) pathways. Pathway enrichments were evaluated by their normalized enrichment score (NES), nominal *P* value, and false discovery rates (FDR).

**Figure 1:**
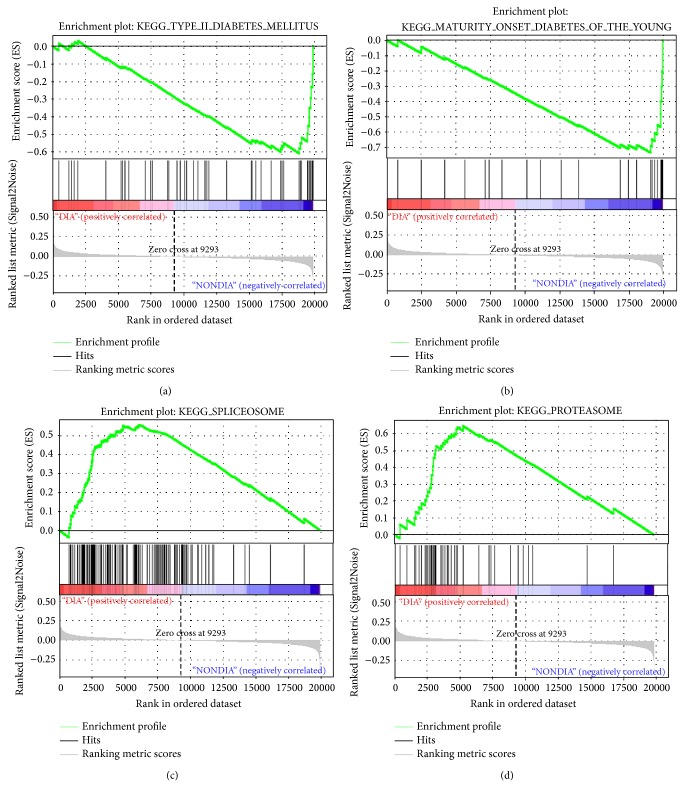
GSEA plot. The analysis was performed against the KEGG database for differential enriched pathways between hyperglycemic and normoglycemic islets. Enrichment plots for the downregulated pathways are shown in graphs (a) and (b) and upregulated pathways are shown in graphs (b) and (c). The *y*-axis represents the value of the ranking metric; the *x*-axis represents the rank for all genes. Bottom: plot of the ranked list of all genes. Top: the enrichment score for the gene set as the analysis walks along the ranked list. The score at the peak of the plot is the enrichment score (ES) for this gene set and those genes appearing before or at the peak are defined as core enrichment genes in this gene set. Lower levels of expression are represented in shades of blue and higher expression is represented in red.

GSEA identified 4 pathways (*P* < 0.05) which were downregulated in the hyperglycemic islets compared to normoglycemic islets (Table 2). At FDR < 25%, only two pathways (T2DM and MODY) were shown to be significant (Table 2 and Figures 1(a)-1(b)). On the other hand, six enriched pathways were upregulated in the hyperglycemic donors (*P* < 0.05), while at FDR < 25% only two pathways (proteasome and spliceosome) were significant (Table 2 and Figures 1(c)-1(d)).

**Figure 2:**
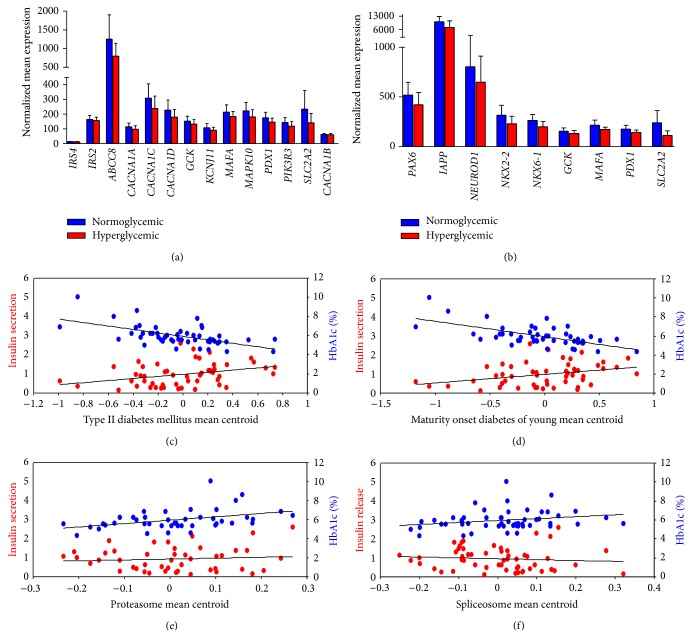
Genes differentially expressed in T2DM and MODY pathways. Gene expression analysis of genes in the T2DM and MODY pathway showed that 14 out of the 45 genes of the T2DM (a) pathway (*P* < 0.05) and 9 out of 24 genes of MODY (b) (*P* < 0.05) have lower expression in hyperglycaemic compared to normoglycemic donors. (c) Correlation of mean centroid of the 14 downregulated genes in T2DM pathway showed positive correlation with insulin secretion (*R* = 0.33; *P* = 0.01) and negative correlation with HbA1c level (*R* = −0.57; *P* = 0.00001). (d) Correlation of mean centroid of the 9 downregulated genes in MODY pathway showed positive correlation with insulin secretion (*R* = 0.31; *P* = 0.01) and negative correlation with HbA1c level (*R* = −0.59; *P* = 0.000006). (e) Correlation of mean centroid of the 31 upregulated genes in proteasome pathway showed no correlation with insulin secretion (*R* = −0.05; *P* = 0.7) and positive correlation with HbA1c level (*R* = 0.3; *P* = 0.03). (f) Correlation of mean centroid of the 55 upregulated genes in spliceosome pathway showed no correlation with insulin secretion (*R* = 0.16; *P* = 0.23) and positive correlation with HbA1c level (*R* = 0.26; *P* = 0.06).

Next, we examined individual expression value of the 45 genes in the T2DM and the 24 genes in MODY pathway. We found that 14 out of the 45 genes of the T2DM pathway (31%) and 9 out of the 24 genes of MODY (37.5%) contributed significantly to core enrichment whose expression was lower in hyperglycemic than in normoglycemic donors (Figures 2(a)-2(b)). Also, expression of the 23 genes was significantly reduced in diabetic compared to nondiabetic donors (Table S1 in Supplementary Material available online at http://dx.doi.org/10.1155/2014/237535). Insulin receptor substrate genes (IRS4) were shown to be low/not expressed in human pancreatic islets (Figure 2(a)). Glucokinase (GCK), solute carrier family 2 (facilitated glucose transporter), member 2 (SLC2A2), pancreatic and duodenal homeobox 1 (PDX1), and v-maf musculoaponeurotic fibrosarcoma oncogene homolog A (MAFA) overlapped between core enrichment of the two pathways. Interestingly, plotting the mean centroid of the 14 and 9 downregulated genes in T2DM and MODY pathways showed a positive correlation with insulin secretion and negative correlation with an HbA1c level (Figures 2(c)-2(d)) suggesting that these pathways are involved in regulation of insulin secretion and glycemic status. Mean centroid of the 31 and 55 upregulated genes in the core enrichment of proteasome and spliceosome pathways showed no correlation with insulin secretion but positively correlated with HbA1c level (Figures 2(e)-2(f)). We also analysed the real score of expression and differential expression of genes in the core enrichment of proteasome and spliceosome pathways in normoglycemic versus hyperglycemic and in nondiabetic versus diabetic donors (Tables S2 and S3).

## 4. Discussion

Although several pathways have been implicated in T2D pathogenesis, most of these studies were performed in nonpancreatic tissues. In this study, we used human pancreatic islets obtained from 78 donors. Each donated pancreatic islet was systematically characterized by performing cDNA microarray in addition to measuring insulin response to glucose and glycemic status (HbA1c).

Our data presented additional evidence into the biological processes that differentially were regulated in pancreatic islets from normoglycemic and hyperglycemic donors. The downregulated pathways (T2DM and MODY) in hyperglycemic donors were due to decreased expression of several protein-encoded genes, which indicate a reduction in protein synthesis in pancreatic islets. Recently, Del Rosario et al. reported that regions in promoter of genes involved in T2DM and MODY pathways are more likely to be differentially methylated between diabetic and nondiabetic donors compared to other genes [16]. In this study, the islet expression mean centroid of downregulated genes in T2DM and MODY pathways correlated positively with insulin secretion and negatively with HbA1c level, suggesting that appropriate expression of these genes is required for sufficient insulin secretion and glucose homeostasis.

Notably, most of the downregulated genes have been implicated in diabetes pathogenesis;* SLC2A2* (*Glut2*) is involved in *β*-cell function and insulin secretion [17]. Mice lacking* SLC2A2* showed early diabetes and abnormal postnatal pancreatic islet development [18].* ABCC8* is a regulator of ATP-sensitive K(+) channels and insulin release. A mutation in* ABCC8* was observed in patients with hyperinsulinemic hypoglycemia of infancy [19] and associated with T2D [20].* KCNJ11*, together with ABCC8, regulates transmembrane potential and thereby glucose-stimulated insulin secretion in pancreatic beta-cells. A Glu23Lys polymorphism (E23K) has been associated with T2D and a modest impairment in insulin secretion [21]. In addition, mutation in the gene causes a severe form of neonatal diabetes as well as maturity onset diabetes of the young type 11 (MODY11) [22].* PDX1* is involved in the early development of the pancreas and plays a major role in glucose-dependent regulation of insulin gene expression [23]. Defects in this gene caused maturity onset diabetes of the young type 4 (MODY4) [24].* PAX6*, point mutations in the* PAX6* gene shown to disrupt islet morphology and decreased numbers of *β*, *α*, and PP cells [25]. Also, a mutation in the gene has been shown to cause early-onset diabetes [26].* MAFA* is required for islet beta-cell differentiation and activates the insulin and glucagon promoters [27].* MAFA* functions as a downstream mediator of PAX6 in regulating the specification of insulin and glucagon expressing cells [28].* NEUROD1* is reported to regulate expression of the insulin gene [29], and mutations in this gene result in maturity onset diabetes of the young type 6 (MODY6) [30].* FOXA2* is involved in glucose homeostasis and regulates the expression of genes important for glucose sensing in pancreatic beta-cells and glucose homeostasis [31]. Hence, these data suggest that the downregulation of the 23 genes in T2DM and MODY pathways is a causative for insulin secretion impairment.

Proteasomes are protein complexes with a main function to regulate and degrade unnecessary or damaged proteins by proteolysis, while spliceosome is a complex molecular machine assembled from snRNPs and protein complexes. Splicing is a known process when spliceosome removes introns from a transcribed pre-mRNA. Both of the proteasome and the spliceosome pathways showed upregulation in hyperglycemic donors. The findings are potentially very important as there are several lines of evidence which reported glucose or hyperglycemia to influence proteasome and splicing. Recent studies have shown that high glucose and diabetes affect proteasome activity [32, 33]. The link of hyperglycemia to proteasome raises several questions such as how hyperglycemia can modulate proteasome targeting and activity and whether this modulation occurs in a cell-specific manner. Hribal et al. reported that chronic hyperglycemia impairs insulin secretion by affecting splicing in RIN *β*-cell line and human islet [34]. Osmark et al. reported pronounced tissue-specific differences in the splicing of TCF7L2 with forms containing exons 4 and 15 being the most abundant in islets. The incorporation of exon 4 in islets was shown to correlate positively with HbA1c levels [35]. Also, a short TCF7L2 mRNA variant in subcutaneous fat is associated with hyperglycemia and impaired insulin action in adipose tissue [36]. Although these reports do not prove causality, they suggest an effect of plasma glucose levels on splicing.

There is one confine in our study that must be acknowledged. The study employed whole pancreatic islet; the relative contribution of the transcriptional programs in specific cell types towards the observed gene expression differences cannot be clearly delineated. However, most of the downregulated genes in the core enrichment of T2DM and MODY are more expressed in *β*-cell compared to alpha cells and exocrine cells as shown in recent published RNA-sequencing expression data from sorted endocrine cells [37].

In conclusion, the investigation of gene expression profiles from pancreatic islets can illustrate some of the biological processes related to the regulation of glucose hemostats and diabetes pathogenesis.

## Supplementary Material

Real score of RNA expression and anlysis for T2DM, MODY, Proteosome and Splicesome pathways between normoglycemic vs. hyperglycemic and non-diabetic vs. diabetic donors.

## Figures and Tables

**Table 1:** Characteristics of human pancreatic donors.

	Normoglycemic	Hyperglycemic
*N* (male/female)	58 (34/24)	20 (11/9)
Age (years)	60.9 ± 10.9	64 ± 8.9
BMI	25.4 ± 2.9	28.5 ± 4.5
HbA1c	5.4 ± 0.3	6.9 ± 1.0
Purity	70 ± 16	63 ± 20
Donors with diabetes	0	10

Data represented as mean ± SD.

**Table 2:** List of down- and upregulated pathways in hyperglycemic donors.

	Size	NES	NOM *P* value	FDR *q* value
*Downregulated KEGG pathways *				
KEGG_TYPE_II_DIABETES_MELLITUS	45	−1,898	0	0,077
KEGG_MATURITY_ONSET_DIABETES_OF_THE_YOUNG	24	−1,812	0	0,108
KEGG_OOCYTE_MEIOSIS	108	−1,543	0,02	0,485
KEGG_PROGESTERONE_MEDIATED_OOCYTE_MATURATION	83	−1,453	0,04	0,70
KEGG_SNARE_INTERACTIONS_IN_VESICULAR_TRANSPORT	38	−1,583	0,05	0,610
*Upregulated KEGG pathways *				
KEGG_PROTEASOME	44	2,026	0,005	0,030
KEGG_SPLICEOSOME	125	1,898	0,01	0,075
KEGG_DNA_REPLICATION	36	1,621	0,04	0,821
KEGG_PRIMARY_IMMUNODEFICIENCY	35	1,604	0,02	0,71
KEGG_CYTOKINE_CYTOKINE_RECEPTOR_INTERACTION	251	1,593	0,003	0,619
KEGG_GLYOXYLATE_AND_DICARBOXYLATE_METABOLISM	16	1,529	0,03	0,614

Ranking of the genes set was done using GSEA 2.0.13. NES: normalized enrichment score; NOM: nominal; FDR: false discovery rate.
