# Supplementary material for: Downregulation of Type II Diabetes Mellitus and Maturity Onset Diabetes of Young Pathways in Human Pancreatic Islets from Hyperglycemic Donors
Source: J Diabetes Res. 2014 Oct 14;2014:237535. doi: 10.1155/2014/237535 (PMC4212628; doi:10.1155/2014/237535)
Supplement: Supplementary file 1 — Real score of RNA expression and anlysis for T2DM, MODY, Proteosome and Splicesome pathways between normoglycemic vs. hyperglycemic and non-diabetic vs. diabetic donors. [file 237535.f1.doc]

**Table S1:** Real score of mean expression (A.U.) of the up-regulated genes in the core enrichment of T2DM and MODY pathways in normoglycemic vs. Hyperglycemic and diabetic vs. Nodiabetic.

|  | **Normoglycemic** | | **Hyperglycemic** | |  |  | **Non-diabetic** | | **Diabetic** | |  |
| --- | --- | --- | --- | --- | --- | --- | --- | --- | --- | --- | --- |
|  | **Mean** | **SD** | **Mean** | **SD** | **P-value** |  | **Mean** | **SD** | **Mean** | **SD** | **P-value** |
| **IRS4*** | 12,65164 | 1,334503 | 12,82001 | 1,677284 | N.A |  | 12,72842 | 1,429545 | 12,84135 | 1,778233 | N.A |
| **IRS2** | 162,2147 | 27,00206 | 150,6531 | 19,93647 | 0,03 |  | 160,6765 | 25,05507 | 160,667 | 22,89426 | 0,38 |
| **ABCC8** | 1160,929 | 659,6309 | 996,9993 | 538,9588 | 0,00 |  | 1108,619 | 627,5081 | 1047,511 | 613,4355 | 0,00 |
| **CACNA1A** | 111,9945 | 31,58054 | 103,2461 | 29,2537 | 0,01 |  | 109,6361 | 31,14815 | 103,9239 | 19,10407 | 0,03 |
| **CACNA1D** | 219,8309 | 65,86927 | 192,2916 | 72,01123 | 0,00 |  | 211,7423 | 69,13658 | 196,7402 | 66,24664 | 0,04 |
| **GCK** | 149,4781 | 37,55381 | 137,7721 | 32,29474 | 0,01 |  | 145,8808 | 36,44865 | 138,5464 | 24,34313 | 0,06 |
| **KCNJ11** | 100,999 | 27,84282 | 95,67016 | 29,11647 | 0,01 |  | 99,72169 | 28,27413 | 98,71087 | 32,51933 | 0,01 |
| **MAFA** | 203,6142 | 49,48734 | 200,4996 | 49,29212 | 0,00 |  | 202,1309 | 49,39034 | 200,3511 | 42,54777 | 0,00 |
| **MAPK10** | 215,2355 | 63,61156 | 194,6036 | 48,83159 | 0,01 |  | 209,3236 | 59,73814 | 190,1975 | 52,57106 | 0,52 |
| **PDX1** | 169,5964 | 37,81293 | 158,6702 | 35,35191 | 0,00 |  | 166,1135 | 37,55529 | 160,672 | 31,74163 | 0,00 |
| **PIK3R3** | 139,2655 | 37,11081 | 126,8318 | 33,77501 | 0,01 |  | 135,3241 | 36,72775 | 125,3552 | 31,01973 | 0,10 |
| **SLC2A2** | 208,7503 | 121,9486 | 168,7843 | 99,33549 | 0,00 |  | 194,4945 | 114,7452 | 197,1174 | 128,3916 | 0,01 |
| **CACNA1B** | 61,92747 | 7,506441 | 60,47208 | 7,72169 | 0,05 |  | 61,56282 | 7,613506 | 60,60313 | 8,547841 | 0,01 |
| **PAX6** | 505,7236 | 151,2612 | 461,3979 | 142,6633 | 0,01 |  | 493,4483 | 150,2287 | 449,293 | 103,1056 | 0,33 |
| **IAPP** | 9696,292 | 2908,986 | 9228,081 | 3613,89 | 0,00 |  | 9481,987 | 3176,769 | 9165,072 | 3061,474 | 0,01 |
| **NEUROD1** | 795,2367 | 322,1974 | 702,3624 | 310,4657 | 0,05 |  | 770,3891 | 319,3783 | 680,8645 | 235,2562 | 0,44 |
| **NKX2-2** | 301,8694 | 109,1899 | 265,083 | 92,39113 | 0,00 |  | 290,8235 | 105,7509 | 265,1314 | 85,58379 | 0,09 |
| **NKX6-1** | 251,0378 | 67,41237 | 223,7263 | 67,34273 | 0,00 |  | 242,6999 | 68,881 | 229,3318 | 49,13293 | 0,01 |

Difference in expression levels were analyzed by non-parametic Mann-Whitney test.

* : mean expression belwo background level.

N.A.: not applicable

**Table S2:** Real score of mean expression (A.U.) of the up-regulated genes in the core enrichment of Proteasome pathway in normoglycemic vs. Hyperglycemic and diabetic vs. Nodiabetic.

|  | **Normoglycemic** | | **Hyperglycemic** | |  |  | **Nondiabetic** | | **Diabetic** |  |  |
| --- | --- | --- | --- | --- | --- | --- | --- | --- | --- | --- | --- |
| Gene | Mean | SD | Mean | SD | P-value |  | Mean | SD | Mean | SD | P-value |
| **PSMA1** | 1367,388 | 120,9345 | 1429,979 | 149,5728 | **,123** |  | 1384,093 | 132,9358 | 1439,964 | 150,9038 | **,357** |
| **PSMA3** | 793,6471 | 119,8216 | 821,8296 | 131,3486 | **,291** |  | 808,0583 | 141,215 | 817,386 | 121,0751 | **,806** |
| **PSMA4** | 2030,63 | 258,3172 | 2121,945 | 294,5432 | **,127** |  | 2069,931 | 267,0847 | 2064,081 | 251,4968 | **,601** |
| **PSMA5** | 523,447 | 92,76489 | 545,9219 | 101,3755 | **,350** |  | 532,0682 | 98,12543 | 562,6968 | 95,15587 | **,365** |
| **PSMA6** | 1150,25 | 240,294 | 1202,821 | 202,9216 | **,123** |  | 1157,703 | 244,2243 | 1203,17 | 158,071 | **,282** |
| **PSMA7** | 634,5858 | 88,26718 | 664,0368 | 85,42838 | **,208** |  | 640,0294 | 83,17116 | 663,7728 | 96,67062 | **,623** |
| **PSMB1** | 825,7011 | 141,5575 | 862,7639 | 140,5997 | **,123** |  | 835,9188 | 147,3684 | 831,495 | 84,8576 | **,499** |
| **PSMB2** | 1134,925 | 130,4112 | 1239,445 | 158,7443 | **,009** |  | 1155,053 | 152,3388 | 1198,285 | 101,6477 | **,136** |
| **PSMB3** | 1785,444 | 237,7937 | 1907,166 | 234,4371 | **,027** |  | 1811,324 | 234,4807 | 1853,314 | 282,5531 | **,623** |
| **PSMB4** | 2630,06 | 193,2016 | 2696,246 | 344,7699 | **,623** |  | 2639,903 | 250,4411 | 2646,697 | 286,7229 | **,591** |
| **PSMB6** | 1104,595 | 115,7091 | 1199,121 | 169,9162 | **,012** |  | 1127,178 | 132,6902 | 1172,211 | 181,4741 | **,333** |
| **PSMB7** | 1249,82 | 224,9081 | 1339,701 | 188,324 | **,043** |  | 1285,605 | 216,1064 | 1243,012 | 162,5097 | **,678** |
| **PSMD3** | 676,2483 | 99,02226 | 718,6441 | 82,86453 | **,072** |  | 683,1517 | 96,95151 | 713,5777 | 51,13689 | **,162** |
| **PSMC4** | 582,9772 | 62,75508 | 639,5202 | 71,22566 | **,005** |  | 592,9494 | 68,80296 | 635,2115 | 62,97921 | **,063** |
| **PSMC5** | 933,2475 | 117,3175 | 969,9911 | 126,7127 | **,310** |  | 945,0851 | 126,5335 | 940,4213 | 85,45875 | **,927** |
| **PSMC6** | 321,7813 | 58,56822 | 319,2364 | 68,20793 | **,497** |  | 317,6555 | 62,33087 | 336,4663 | 43,79044 | **,357** |
| **PSMD6** | 1072,073 | 116,4138 | 1132,716 | 136,5852 | **,070** |  | 1087,169 | 135,3871 | 1111,495 | 93,7574 | **,390** |
| **PSMD14** | 1031,614 | 156,4883 | 1097,828 | 149,4316 | **,094** |  | 1047,02 | 163,5904 | 1083,398 | 109,0642 | **,373** |
| **PSMD11** | 952,3479 | 140,7447 | 1033,209 | 109,0673 | **,005** |  | 971,3667 | 140,0488 | 1019,081 | 92,30561 | **,124** |
| **PSMD13** | 335,9695 | 43,67849 | 351,6953 | 47,47506 | **,168** |  | 343,9386 | 49,83908 | 345,316 | 45,84949 | **,770** |
| **PSME3** | 686,2568 | 77,08057 | 764,7944 | 88,81867 | **,001** |  | 700,6192 | 86,63694 | 775,7248 | 96,0013 | **,025** |
| **PSMC1** | 1122,481 | 211,6004 | 1180,908 | 195,0546 | **,133** |  | 1145,546 | 213,295 | 1119,213 | 98,31424 | **,806** |
| **PSMD12** | 532,7687 | 76,42929 | 558,4863 | 79,91485 | **,105** |  | 536,2645 | 73,7927 | 566,6638 | 72,41518 | **,202** |
| **PSMC2** | 1578,854 | 148,3244 | 1625,182 | 198,3095 | **,549** |  | 1597,876 | 157,0334 | 1594,424 | 182,8882 | **,667** |
| **PSMC3** | 588,3599 | 79,33911 | 632,9816 | 77,4603 | **,030** |  | 605,0735 | 82,5836 | 609,6492 | 85,01177 | **,770** |
| **PSMD7** | 1413,445 | 252,308 | 1494,166 | 242,4219 | **,133** |  | 1431,425 | 257,5702 | 1444,032 | 118,9407 | **,365** |
| **PSMD4** | 249,0689 | 27,42193 | 259,2249 | 21,87464 | **,075** |  | 252,8276 | 27,51505 | 245,9292 | 14,65562 | **,519** |
| **PSME2** | 220,288 | 36,50692 | 237,6781 | 21,96347 | **,040** |  | 225,5968 | 33,84119 | 229,8342 | 24,04184 | **,770** |
| **PSME1** | 576,5801 | 87,81792 | 603,1758 | 84,85331 | **,181** |  | 588,5723 | 86,02812 | 573,5175 | 86,57593 | **,701** |
| **PSME4** | 324,5112 | 44,08977 | 335,396 | 52,17566 | **,514** |  | 325,3895 | 47,83888 | 338,1763 | 44,18631 | **,601** |
| **POMP** | 859,7348 | 119,0571 | 901,7354 | 122,8011 | **,189** |  | 875,1674 | 123,8676 | 871,999 | 123,4328 | **,842** |

Difference in expression levels were analyzed by non-parametic Mann-Whitney test.

**Table S3:** Real score of mean expression (A.U.) of the up-regulated genes in the core enrichment of Splicesome pathway in normoglycemic vs. Hyperglycemic and diabetic vs. Nodiabetic.

|  | **Normoglycemic** | | **Hyperglycemic** | |  |  | **Non-diabetic** | | **Diabetic** |  |  |
| --- | --- | --- | --- | --- | --- | --- | --- | --- | --- | --- | --- |
|  | **Mean** | **SD** | **Mean** | **SD** | **P-value** |  | **Mean** | **SD** | **Mean** | **SD** | **P-value** |
| **SNRPE** | 713,3171 | 120,1203 | 777,2083 | 133,5133 | **,014** |  | 729,3112 | 132,3899 | 785,036 | 128,4854 | **,113** |
| **SNRPF** | 132,935 | 26,60508 | 143,2098 | 33,29931 | **,244** |  | 137,3241 | 31,97665 | 130,2254 | 21,39006 | **,634** |
| **SNRPD1** | 200,9044 | 29,4573 | 217,7982 | 31,47423 | **,031** |  | 203,0508 | 31,13578 | 217,8231 | 27,40222 | **,124** |
| **ISY1** | 180,8704 | 23,94951 | 192,4759 | 27,21953 | **,070** |  | 183,0837 | 26,18375 | 188,221 | 26,65558 | **,842** |
| **PPIL1** | 179,6109 | 36,55859 | 198,2487 | 32,72514 | **,027** |  | 181,0087 | 35,53154 | 203,7546 | 23,92727 | **,029** |
| **EFTUD2** | 431,5665 | 60,04351 | 501,784 | 84,02839 | **,001** |  | 444,2564 | 76,77856 | 512,7498 | 69,67922 | **,015** |
| **THOC1** | 186,745 | 43,38979 | 192,9463 | 38,59626 | **,364** |  | 184,1325 | 42,87373 | 200,772 | 34,19402 | **,162** |
| **SNRPA1** | 37,36511 | 25,29294 | 29,34987 | 12,12464 | **,505** |  | 35,43311 | 22,69769 | 31,53691 | 13,93279 | **,975** |
| **NCBP2L** | 13,68836 | 2,388635 | 14,44568 | 2,915084 | **,393** |  | 14,0363 | 2,815787 | 14,31083 | 2,826435 | **,735** |
| **MAGOH** | 253,9114 | 35,51252 | 269,8666 | 38,24898 | **,053** |  | 256,3955 | 35,02286 | 269,4597 | 39,3527 | **,225** |
| **RBM17** | 154,3144 | 14,12923 | 162,4026 | 18,61355 | **,049** |  | 156,8591 | 17,21805 | 158,4124 | 12,67342 | **,591** |
| **PRPF38B** | 240,3732 | 44,91591 | 248,2666 | 45,92249 | **,400** |  | 236,0246 | 45,23501 | 265,8703 | 45,52613 | **,063** |
| **SNRPB** | 383,2962 | 74,08168 | 414,1632 | 63,8765 | **,049** |  | 387,9864 | 70,70766 | 411,3149 | 71,34159 | **,282** |
| **SNRPD3** | 934,5916 | 73,1064 | 989,5607 | 69,67603 | **,010** |  | 938,362 | 78,09158 | 977,1306 | 76,0534 | **,202** |
| **DHX15** | 927,5603 | 85,2871 | 1024,436 | 134,5115 | **,000** |  | 933,6244 | 103,6984 | 1081,304 | 106,4929 | **,000** |
| **HSPA6** | 105,0593 | 114,0295 | 97,98642 | 77,29846 | **,877** |  | 95,83286 | 94,97311 | 111,5487 | 106,6219 | **,262** |
| **RBMX** | 566,9053 | 109,282 | 625,5803 | 106,2282 | **,024** |  | 575,8209 | 114,5926 | 627,8464 | 69,21456 | **,059** |
| **SNRPB2** | 479,9359 | 53,83464 | 500,5816 | 64,63322 | **,148** |  | 486,6044 | 61,56649 | 499,311 | 57,01222 | **,398** |
| **THOC3** | 307,0537 | 60,6843 | 319,301 | 71,26203 | **,447** |  | 307,2082 | 58,74555 | 305,5095 | 94,77363 | **,667** |
| **LSM6** | 62,55603 | 11,87686 | 62,78654 | 7,667669 | **,505** |  | 61,78652 | 10,46612 | 64,11019 | 8,949535 | **,373** |
| **SNRPC** | 317,46 | 53,32769 | 342,3322 | 55,20846 | **,070** |  | 329,0119 | 56,2 | 318,2813 | 49,30231 | **,612** |
| **PLRG1** | 1363,471 | 136,4718 | 1442,58 | 192,3118 | **,160** |  | 1379,595 | 158,2753 | 1415,843 | 185,6634 | **,951** |
| **HNRNPM** | 1486,673 | 277,5148 | 1670,154 | 324,6202 | **,020** |  | 1518,378 | 315,6144 | 1663,488 | 173,295 | **,075** |
| **BUD31** | 247,6807 | 46,02014 | 258,7193 | 41,1558 | **,199** |  | 252,274 | 47,56553 | 253,4377 | 40,25048 | **,690** |
| **SNRPA** | 203,7179 | 30,51241 | 231,6385 | 27,36711 | **,001** |  | 209,1728 | 31,85467 | 234,0549 | 26,94425 | **,026** |
| **SNRNP40** | 317,0524 | 55,7011 | 346,8211 | 77,32198 | **,160** |  | 322,3152 | 58,78961 | 346,1605 | 88,16972 | **,656** |
| **PPIH** | 269,8431 | 49,5354 | 275,1278 | 53,36233 | **,752** |  | 274,0983 | 50,97729 | 258,5914 | 39,70199 | **,282** |
| **CCDC12** | 461,8219 | 54,62957 | 471,9428 | 67,53084 | **,962** |  | 468,3005 | 60,661 | 433,9828 | 35,14828 | **,082** |
| **CTNNBL1** | 165,0615 | 17,02892 | 177,188 | 24,45176 | **,030** |  | 167,0167 | 20,24926 | 173,8971 | 18,87269 | **,255** |
| **TCERG1** | 190,8935 | 42,61584 | 201,6576 | 47,24915 | **,316** |  | 190,393 | 42,97015 | 201,3022 | 39,07112 | **,255** |
| **SF3A3** | 549,5164 | 74,00302 | 598,5778 | 82,92248 | **,049** |  | 555,5737 | 80,18572 | 596,0267 | 80,80806 | **,213** |
| **PQBP1** | 418,9796 | 51,08361 | 436,1507 | 43,98625 | **,156** |  | 422,7868 | 50,98426 | 438,4648 | 35,61358 | **,202** |
| **U2AF1** | 665,0712 | 83,53865 | 695,7866 | 84,95195 | **,059** |  | 667,6006 | 87,51998 | 700,659 | 54,41291 | **,117** |
| **PRPF4** | 564,4625 | 69,05538 | 598,4217 | 92,69788 | **,228** |  | 563,4416 | 75,6121 | 607,4939 | 87,73319 | **,181** |
| **RBM22** | 385,0872 | 50,09065 | 412,488 | 54,72901 | **,077** |  | 390,5514 | 55,08357 | 396,4884 | 24,05023 | **,701** |
| **LSM2** | 148,0993 | 27,1745 | 150,3007 | 27,68314 | **,691** |  | 150,0165 | 27,90148 | 142,5588 | 17,27758 | **,623** |
| **PRPF38A** | 512,1339 | 74,17121 | 531,3532 | 102,0665 | **,540** |  | 505,4399 | 82,11113 | 558,4782 | 86,72004 | **,067** |
| **PRPF3** | 196,3244 | 39,29613 | 198,6497 | 40,0964 | **,681** |  | 193,3362 | 39,36531 | 209,3418 | 32,94003 | **,107** |
| **PUF60** | 478,3257 | 53,68242 | 517,8599 | 53,3932 | **,004** |  | 485,2264 | 56,25314 | 505,6672 | 47,52606 | **,202** |
| **TRA2B** | 923,0939 | 99,85861 | 968,73 | 96,3112 | **,049** |  | 927,1276 | 102,0328 | 979,7354 | 88,41161 | **,085** |
| **RBM8A** | 71,827 | 15,60075 | 69,53793 | 14,11542 | **,783** |  | 72,45308 | 14,68402 | 65,19909 | 16,79425 | **,402** |
| **PRPF40A** | 608,1716 | 70,27338 | 631,2728 | 104,0872 | **,194** |  | 613,2946 | 87,00071 | 618,6667 | 58,90314 | **,866** |
| **DDX23** | 458,7844 | 63,32811 | 487,8762 | 78,46446 | **,039** |  | 463,5354 | 76,58355 | 488,3599 | 43,97035 | **,282** |
| **RBM25** | 325,3951 | 52,19016 | 342,1287 | 60,82045 | **,176** |  | 326,7564 | 52,23919 | 354,7957 | 51,56231 | **,065** |
| **SF3B14** | 589,6778 | 74,71846 | 600,4227 | 73,50636 | **,336** |  | 594,7441 | 76,69062 | 595,2922 | 74,69589 | **,951** |
| **DDX46** | 274,6208 | 41,54881 | 291,6798 | 48,7352 | **,223** |  | 278,462 | 46,0272 | 283,8994 | 24,81911 | **,656** |
| **LSM7** | 183,9105 | 25,83788 | 189,3691 | 34,69537 | **,595** |  | 187,6128 | 30,6718 | 183,2934 | 21,87114 | **,794** |
| **DDX42** | 455,6061 | 56,97642 | 467,9917 | 88,38052 | **,423** |  | 452,0225 | 64,53608 | 484,1891 | 81,10726 | **,225** |
| **LSM5** | 95,74532 | 19,09918 | 92,51944 | 16,20393 | **,701** |  | 96,71784 | 19,86997 | 90,36618 | 16,90823 | **,529** |
| **U2AF2** | 307,3687 | 33,54049 | 326,2353 | 43,81558 | **,072** |  | 306,5435 | 35,51885 | 347,4091 | 45,9233 | **,005** |
| **SF3B4** | 158,7936 | 17,08919 | 172,8429 | 19,09615 | **,007** |  | 161,9149 | 17,33577 | 171,397 | 20,83534 | **,149** |
| **AQR** | 383,349 | 65,79932 | 386,8759 | 62,9396 | **,681** |  | 377,3069 | 66,46194 | 406,4049 | 60,24171 | **,162** |
| **DHX16** | 217,9614 | 23,99843 | 225,1105 | 30,13003 | **,185** |  | 217,6912 | 26,82838 | 230,3955 | 22,57979 | **,110** |
| **LSM3** | 466,8411 | 89,13004 | 482,8445 | 91,15123 | **,540** |  | 481,8715 | 92,95993 | 450,8358 | 97,02295 | **,186** |
| **DDX5** | 2059,468 | 284,076 | 2103,866 | 237,8452 | **,447** |  | 2056,921 | 279,9836 | 2129,018 | 241,5655 | **,424** |

Difference in expression levels were analyzed by non-parametic Mann-Whitney test
